# Reduced immune response to SARS-CoV-2 infection in the elderly after 6 months

**DOI:** 10.3389/fimmu.2025.1596065

**Published:** 2025-05-09

**Authors:** Qin Luo, Qinqin Song, Yan Li, Kexin Zong, Ti Liu, Junming He, Guoyong Mei, Haijun Du, Zhiqiang Xia, Mi Liu, Juan Song, Chen Gao, Dong Xia, Guangyu Xue, Wenyan Tian, Yinli Qu, Zengqiang Kou, Zhongjun Dong, Jun Han

**Affiliations:** ^1^ National Key Laboratory of Intelligent Tracking and Forecasting for Infectious Diseases, National Institute for Viral Disease Control and Prevention, Chinese Center for Disease Control and Prevention, Beijing, China; ^2^ Shandong Center for Disease Control and Prevention, Shandong Provincial Key Laboratory of Intelligent Monitoring, Early Warning, and Prevention and Control of Infectious Diseases, Shandong Institute of Preventive Medicine, Jinan, China; ^3^ Department of Infectious Diseases, The First Affiliated Hospital of Xi’an Jiaotong University, Xi’an, China; ^4^ Beijing Tsinghua Changgung Hospital, School of Clinical Medicine, Tsinghua University, Beijing, China; ^5^ The First Affiliated Hospital of Anhui Medical University and Institute of Clinical Immunology, Anhui Medical University, Hefei, China; ^6^ State Key Laboratory of Membrane Biology, School of Medicine and Institute for Immunology, Tsinghua University, Beijing, China

**Keywords:** omicron BA.5, elderly, neutralizing antibody, adaptive immunity, T cell

## Abstract

**Objectives:**

To evaluate the immune persistence and cross-immune response of elderly individuals after Omicron BA.5 infections.

**Method:**

The neutralizing antibodies against WT, BA.5, XBB.1 and EG.5 strains were analyzed. The T/B-cell subsets’ responses were tested through intracellular cytokine staining and flow cytometry.

**Results:**

The neutralizing antibodies titers against WT and BA.5 strain, remaining high level for at least 6 months, were higher than that of both XBB.1 and EG.5 variants. The neutralizing antibodies of WT, BA.5, XBB.1, and EG.5 strains in the elderly were slightly lower than those in middle-age. The memory B cells decreased rapidly in the elderly, and Tfh, Th17 cells of the elderly continued to increase for only 3 months, while Tfh and Th17 cells increased in the middle-aged for over 6 months. For the elderly, after peptide stimulation, unswitched/switched memory B cells decreased, while double negative B cells displayed higher proliferation. The proportions of both naïve and Temra cells in CD4^+^ and CD8^+^ T cells declined, whereas those of Tcm and Tem cells elevated. In the meantime, both CD69^+^ and CD38^+^ T cells decreased, but the frequencies of PD-1^+^ and CTLA-4^+^ of CD4^+^ and CD8^+^ T cells showed an increasing trend. The proportions of PD-1^+^ and CTLA-4^+^ cells also increased in older people with long COVID symptoms at 3m post-infection.

**Conclusions:**

Omicron BA.5 infection induced lower neutralizing antibodies against XBB.1 and EG.5 variant. The decrease of memory B cells, CD69^+^ and CD38^+^T cells, as well as the increase of PD-1^+^, CTLA-4^+^ of CD4^+^/CD8^+^T cells and double negative B cells, indicate that sustained immune responses against BA.5 infection may wane more rapidly in elderly populations.

## 1 Introduction

Coronavirus disease 2019 (COVID-19) is an acute respiratory infection caused by severe acute respiratory syndrome coronavirus 2 (SARS-CoV-2). As of March 2025, COVID-19 causes a pandemic with over 776 million cases and at least 7 million deaths (1). To date, Alpha, Beta, Gamma, Delta, and Omicron variants have been classified as a ‘variant of concern’ by the World Health Organization (WHO) on the basis that they exhibit substantially altered transmissibility or immune escape, warranting close monitoring (2, 3). After November 2021, the emergence of the Omicron (B.1.1.529) variant continued to mutate to novel subtypes, resulting in increasing immune escape and risk of reinfection and gradually evolved into the dominant strain in the world (4, 5). In December 2022, there was an outbreak of the Omicron BA.5.2/BF.7 variant in China (6). From May to July 2023, the Omicron XBB variant with stronger immune evasion reached its peak of prevalence (4). And the EG.5 variant reached its peak of prevalence in November 2023 (7). Since early 2022, Omicron variants replace Delta and Alpha, with all currently circulating SARS-CoV-2 strains belonging to Omicron sub-lineages (2). In addition, neutralizing antibodies (Nab) may provide evidence for protection against reinfection in elderly people, although immunosenescence may not produce an adequate protective response (8, 9). Several clinical studies show that poor antibody response is one of the risk factors for infection in the elderly, which is related to the decline of vaccine efficiency and the serious consequences of COVID-19 (10–13). Some studies show that the antibody responses of elderly participants after Omicron BA.5 infection and XBB reinfection may be affected by vaccine type and comorbidities (14–16). The continuous evolution of Omicron subvariants has led to substantial reductions in vaccine effectiveness (17). Given the ongoing evolution of the SARS-CoV-2 virus and the emergence of new variants, it is essential to conduct longitudinal studies on immune persistence in the elderly. This research is crucial for understanding how better to protect this vulnerable group from SARS-CoV-2 variants infection again.

The immunological memory is one of the important features of adaptive immunity. Studies of convalescent COVID-19 patients show that the level of SARS-CoV-2-specific antibodies gradually decreases over time (18–20). Multiple evidence suggests that T-cell responses are associated with the reduction of symptoms of COVID-19 disease (21–23). Both natural infection and vaccination can induce the population’s immunity against COVID-19. However, previous studies find that compared with the young, the elderly have poor T-cell responses to SARS-CoV-2 caused by vaccination or infection (24). The patients over 65 years old have a very uncoordinated SARS-CoV-2 specific immune response compared with young patients, in which there is a reduction coordination of between CD4^+^ and CD8^+^T cell responses, the loss coordination between CD4^+^T cells and antibody response, and changes in correlation between inflammatory cytokines and CD4^+^T cells, CD8^+^T cells, and antibody response, especially, in patients aged ≥75 years old (21). SARS-CoV-2 infection induces specific T cell responses to various viral antigens, but the diversity of target antigen repertoire for long-lived memory T cells specific for SARS-CoV-2 may decline with age (25).

Several studies have reported report the immune responses and epidemiological features after Omicron BA.5 infection in the general population (6, 16, 26). However, it is necessary to study the immune persistence of the elderly population and determine the characteristics of T-cell immune responses that prevent reinfection in the elderly. Thus, a longitudinal study of immunity response to SARS-CoV-2 was conducted in the elderly population of Shandong, China.

## 2 Methods

### 2.1 Study design and participants

**Figure 1:**
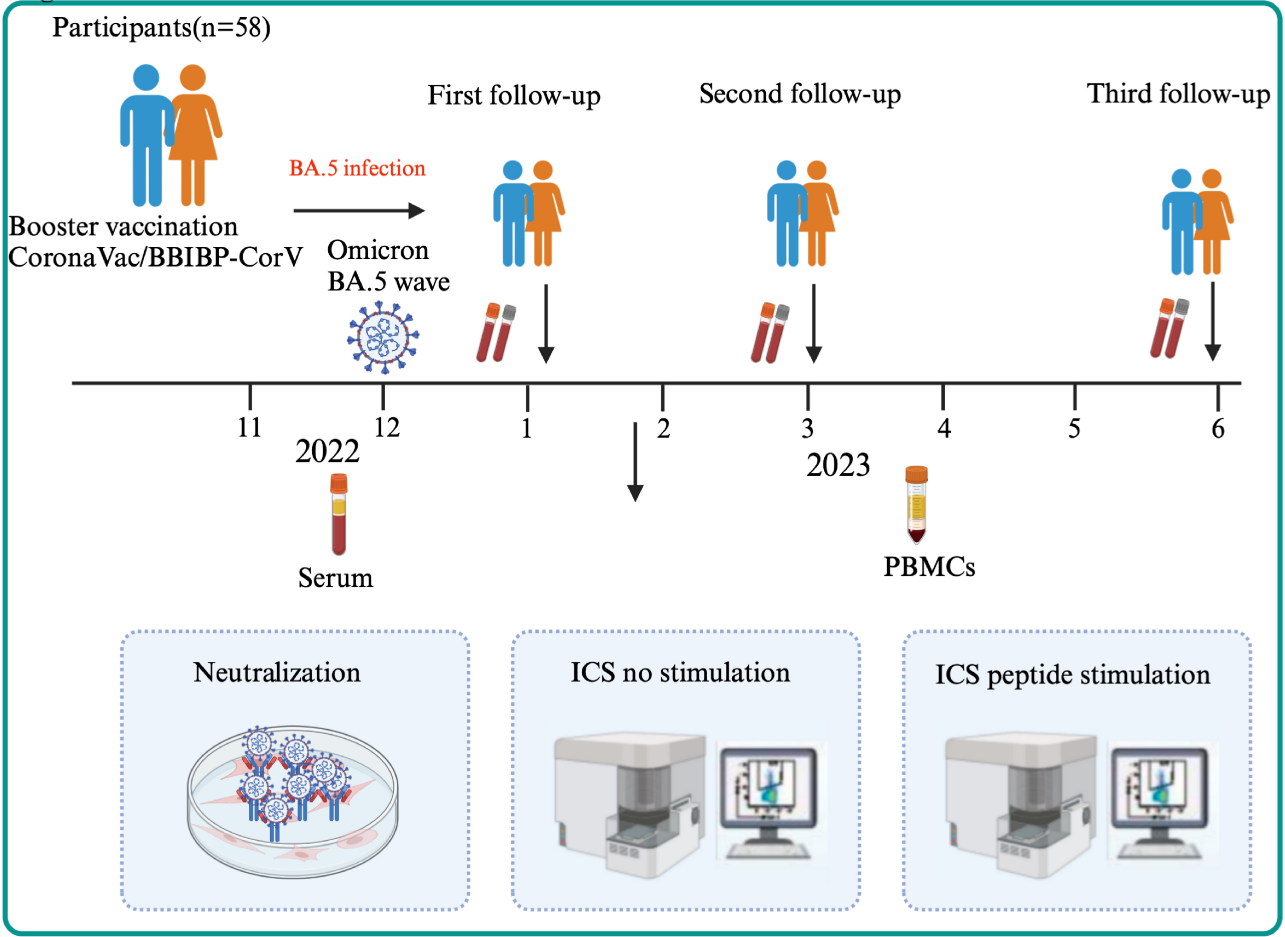
The flow chart of the study design. A total of 58 participants were enrolled in three visits (1m, 3m, 6m after BA.5 infection) followed up longitudinally in Shandong, China in 2023. After serum and PBMCs of participants were collected, Nab titer of WT strain, BA.5, XBB.1 and EG.5 variant and the cellular immune were detected through ICS using no peptide stimulation for all participants or peptide stimulation for the elderly.

58 participants infected with Omicron BA.5 in December 2022 were recruited, and three follow-up visits were carried out in January, March, and June 2023 (**Figure 1**) in Shandong, China. Among the 58 participants were non-hospitalized (classified as mild or moderate cases), including 9 asymptomatic individuals and 49 symptomatic individuals who recovered without requiring hospitalization. After venous blood was collected from all participants, serum, plasm, and peripheral blood mononuclear cells (PBMCs) were isolated. Before testing, PBMCs were frozen and stored in serum-free cell-freeing media (Aoqing Biotechnology Co., Ltd., China) at -80°C. This ethics was approved by the Ethics Committee of National Institute for Viral Disease Control and Prevention, China CDC and written informed consent was provided by the participants or their guardians before sample collection (No. IVDC2025-003).

### 2.2 Neutralizing antibody assays

The NAbs to SARS-CoV-2 were detected by the microneutralization tests under Vero cell using the SARS-CoV-2 protype strain and variants BA.5, EG.5.1, XBB.1.1 according to previous reports (27), the neutralization assays of SARS-CoV-2 were carried out under Vero cells with DMEM supplemented containing penicillin G (100 U/ml), streptomycin (100 mg/ml), 2 mM L-glutamine, and 10% heat-inactivated fetal bovine serum (FBS). All cell cultures were maintained in 5% CO2 at 37°C. The steps of the neutralization test were briefly described as follows: 100 μL of serum diluted in a 1:2 series (starting at 1:4) were added to each well of 96-well plates. Then, each well of 96-well plates was seeded with 20,000 cells in 100 μL of DMEM. Subsequently, 50μL of cell culture medium containing 100 TCID50 Wild type, BA.5, XBB.1 and EG.5 variant were added in 96-well plates under 37°C and the cytopathic effect (CPE) was observed after 5-day incubation.

### 2.3 Evaluation of T/B cells response using flow cytometry

PBMC were washed in PBS with 1% heat-inactivated FBS (Gibco), stained with Live/Dead fix blue (Life Technologies, 1:500), and incubated for 30 minutes at 4°C. Cells were washed in PBS+1% FBS and incubated for 30 minutes at 37°C with CCR7-BV421 (Biolegend) and CD185-BV750 (Biolegend). Then, Cells were washed in PBS+1% FBS and incubated for 30 minutes to 1h at 4°C sheltered from light with an antibody cocktail including: CD8-BUV805 (BD), CD19-Spark NIR685 (Biolegend), CD27-BV480 (BD), CD38-APC/Fire810 (Biolegend), CD56-BUV737 (BD), CD16, CD45RA-BUV395 (BD), CD25-PE-Fire700 (Biolegend), CD127-APC-R700 (BD). Next, Cells were washed in PBS+2% FCS and permeabilized for 50 minutes in 4°C in 100μL of Transcription Factor Buffer Set (BD Biosciences). After washing in PermWash (100μL 1×perm/wash buffer), antibodies were formulated using 1×perm/wash buffer and incubated for 30 minutes at 4°C, including IL-17A-PE(Biolegend), CD4-BUV496 (BD), and CD3-BV510 (Biolegend). After washing in 1×perm/wash buffer and cell resuspension solution in 2% PFA, flow cytometry was performed using Cytek Aurora flow cytometry.

After PBMC of the elderly were collected in 3m and 6m of post-infection (pi), ICS was performed using stimulation of spike peptide pools of BA.5 (Qiangyao Biotechnology Co., Ltd., China). Briefly, cryopreserved PBMCs were thawed at 37°C and immediately washed in PBS, the suspension was performed using CTL medium containing nuclease (Benzonase^®^, 1:5000), and the cells were recovered at 37°C for 3 to 4h and counted. Cells were cultured for 6 hours in the 5 μL PMA+Ionomycin (Dakewe), DMSO, and spike peptide pools of the BA.5 variants (5μg/mL) in 96-wells U-bottom plates at 1x10^6^ PBMC per well, respectively. And add eBioscience^TM^ Brefeldin A Solution per well blocker. Stimulation with an equimolar amount of DMSO was performed as a negative control, and PMA+Ionomycin was included as a positive control. Then, dyeing of PBMC treatment as described above. Extracellular antibody cocktail including CD19-Spark NIR685 (Biolegend), CD25-PE-Fire700 (Biolegend), CD366-PE/Dazzle594 (Biolegend), CD152-PE (Biolegend), CD45RA-BUV395 (BD), CD20-BV570 (Biolegend), CD279-BV785 (Biolegend), CD38-APC/Fire810 (Biolegend), CD27-BV480 (BD), IgD-PE/Cyanine5 (Biolegend), CD8-BUV805 (BD), CD56-BUV737 (BD), CD69-BUV661 (BD). Intracellular antibody cocktail including Perforin-FITC (BD), CD4-BUV496 (BD), Granzyme B-eFluo 450 (Thermo) and CD3-BV510 (Biolegend). After washing in 1×perm/wash buffer and cell resuspension solution in 2% PFA, flow cytometry was performed using Cytek Aurora flow cytometry.

### 2.4 Statistical analysis

The demographic characteristics of the participants were presented as the median (IQR) for continuous variables and the absolute values and percentages for categorical variables. Nab were presented as the geometric mean titers (GMTs) with 95% confidence intervals (CIs). The difference between groups was examined by a Wilcoxon matched-pairs signed rank test or Mann-Whitney U-test as appropriate. A two-sided P-value < 0.05 was considered statistically significant. The statistical analysis was conducted using GraphPad Prism 9.0 (GraphPad Software, San Diego, USA) and IBM SPSS Statistics 26 (SPSS Inc., Chicago, USA).

## 3 Results

### 3.1 The epidemiological characteristics of participants

A total of 58 participants were enrolled in the study, their median age was 60.5 years old (age range: 34–86 years old) (**Table 1**), and the male to female ratio was 1:1.30. Among them, 53.44% of the population was over 60 years old, with a male to female ratio of 1:1.38. All individuals received SARS-CoV-2 vaccine. 96.55% (56/58) of people received a booster dose of inactivated vaccine. Among them, 89.66% (52/58) of people received at least one booster of CoronaVac or BBIBP-CorV vaccine. The first dose of inactivated vaccines was primarily administered in 2021(96.55%, 56/58), and the second dose was also mainly administered in 2021(93.10%, 54/58), for the third vaccination, 55.36% (31/56) individuals received it in 2021 and 44.64% (25/56) in 2022. Additionally, 39.66% (n=23) of the participants suffered from underlying diseases, of which hypertension, diabetes, or coronary heart disease were the most common comorbidities. Among the participants, 49 were symptomatic and 9 were asymptomatic according to a questionnaire survey. The common symptoms were fever (87.76%), cough (75.55%), fatigue (73.47%), expectoration (71.43%), and sore throat (65.31%).

**Table 1:** The epidemiological characteristics of participants.

Characteristics	Participants (n = 58)
(median, IQR)	60.5 (48-70)
Sex (%)	
Male	25 (43.10)
Female	33 (58.90)
Comorbidities (%)	
Yes	23 (39.66)
No	35 (60.34)
Omicron BA.5 infection (%)	
asymptomatic	9 (15.52)
symptomatic	49 (84.48)
Symptomatic infection(n=49)	
Fever	43 (87.76)
Cough	38 (75.55)
Fatigue	36 (73.47)
Expectoration	35 (71.43)
Sore throat	32 (65.31)
Pain in the lower back and limbs	30 (61.22)
Headache	26 (53.06)
Dizzy	22 (44.90)
Chest distress	21 (42.86)
Diarrhea	12 (24.49)
Nausea or vomiting	9 (18.37)
Booster vaccination (n=52)	
CoronaVac	22 (42.31)
BBIBP‐CorV	30 (57.69)

### 3.2 Changes of Nab titers after 6 months of BA.5 infection

**Figure 2:**
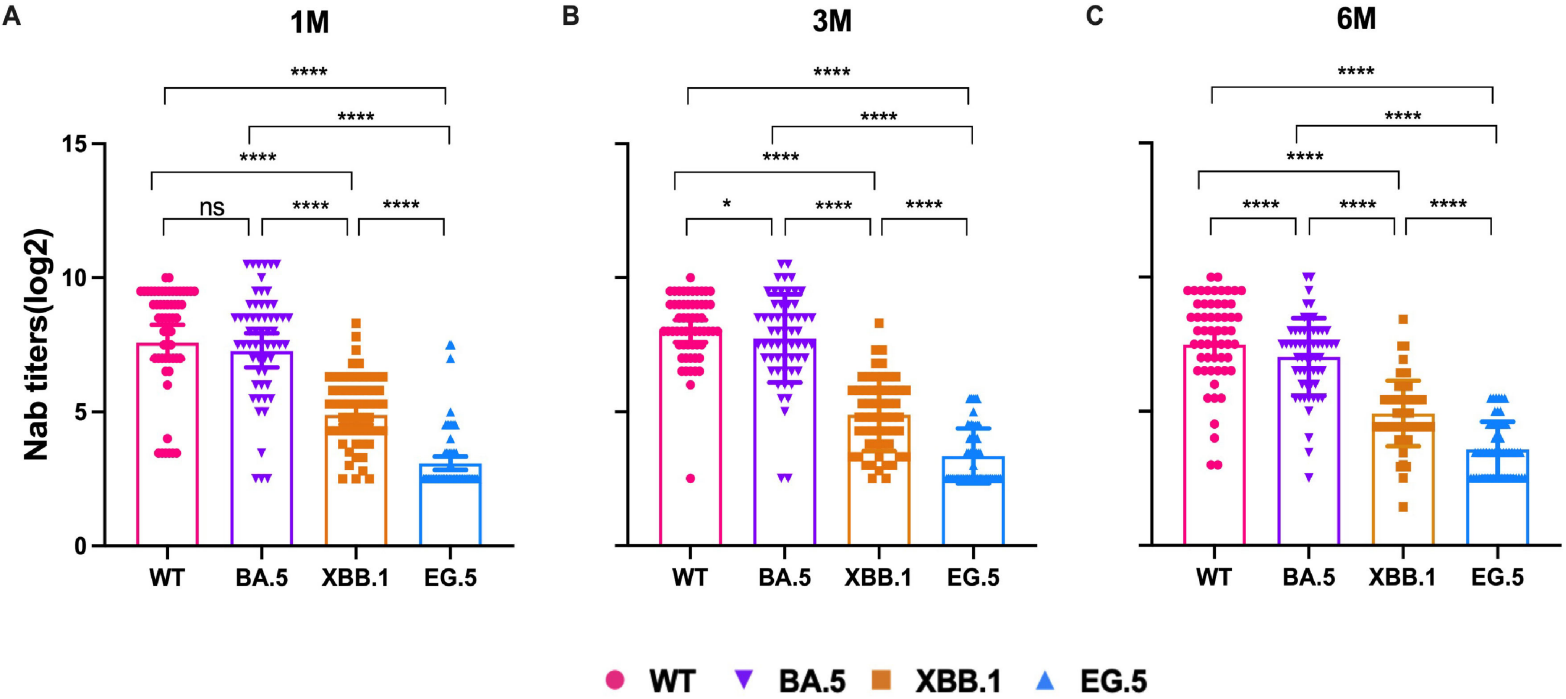
NAb titers of WT and Omicron BA.5, XBB.1, EG.5 variants in 1m, 3m and 6m pi, respectively. NAb titers changes in the 58 participants in 1m **(A)**, 3m **(B)**, and 6m **(C)** after BA.5 infection. The bar indicates geometric mean titers (GMT) with a 95% confidence interval (CI). A Wilcoxon matched-pairs signed rank test was performed in this analysis and *P* < 0.05 was considered statistically significant. *P < 0.05, **P < 0.01, ***P < 0.001, ****P < 0.0001. ns represents no statistical significance.

To evaluate the changes in Nab titers in individuals infected with the Omicron BA.5 variant, a follow-up study was conducted involving 58 participants. Serum samples were collected at three time points: the 1^st^ month, the 3^rd^ month, and the 6^th^ month pi. The Nab titers were tested against the WT, BA.5, XBB.1, and EG.5 strains, respectively. The Nab titers against the WT strain were the highest at each time point, followed by BA.5, XBB.1, and EG.5 variants (**Figure 2**). GMTs of WT strain was 238.76 (95% CI, 167.11-341.15), 278.49 (95% CI, 221.8-349.67), and 208.29 (95% CI, 153.81-282.09) in 1m, 3m and 6m pi, and the GMTs of BA.5 variant were 194.41 (95% CI, 135.52-278.89), 212.82 (95% CI, 157.67-287.24), and 130.26 (95% CI, 100.16-169.41). The XBB.1 variant was 33.62 (95% CI, 26.40-42.85), 29.54 (95% CI, 22.99-37.97), and 30.16 (95% CI, 24.1-37.75). And there was a lower level of the Nab titers against EG.5 variant. The antibody titers of EG.5 variant at the three time points were 9.48 (95%CI, 7.53-11.93), 10.18 (95%CI, 8.42-12.31), and 11.98 (95%CI, 9.91-14.50) (**Supplementary Figure 1**), respectively. The titers of XBB.1 were remarkably lower than those of BA.5 by 5.78-fold, 7.20-fold, and 4.32-fold in 1m, 3m, and 6m pi, respectively. Whether the elderly or the middle-aged, Nab titers against both WT and BA. 5 strains remained at a high level at each time point, and those of XBB.1 and EG.5 variants were at a relatively low level, especially the EG.5 variant (**Supplementary Figure 2**). But the Nab titers of the WT and BA.5 strains were lower in older people than in the middle-aged (*P*>0.05) (**Supplementary Figure 2**).

**Figure 3:**
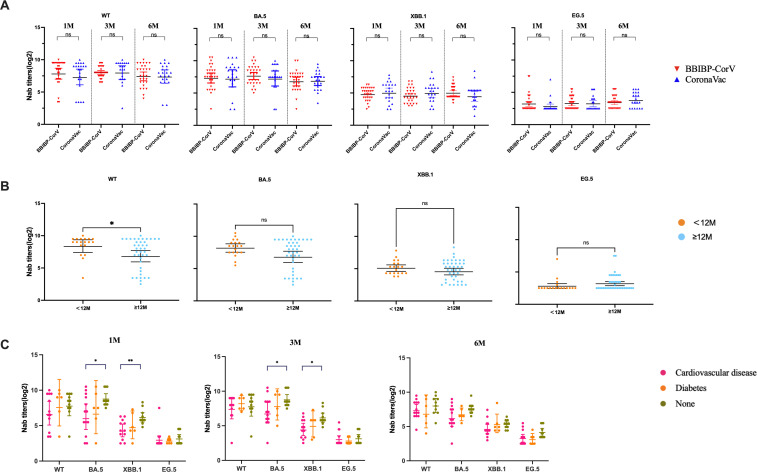
The impact of vaccination and comorbidities on Nab levels of participants. **(A)** Comparison of Nab titers against WT strain, BA.5, XBB.1 and EG.5 variants in patients infected with SARS-CoV-2 after receiving BBIBP-CorV and CoronaVac vaccines. **(B)** Comparison of Nab levels against the WT strain, BA.5, XBB.1 and EG.5 variants between individuals with SARS-CoV-2 inactivated vaccine booster-infection intervals of <12 months and ≥12 months. **(C)** Comparison of Nab levels against the WT strain, BA.5, XBB.1, and EG.5 variants between elderly individuals with comorbidity and those without comorbidity. The bar indicates the GMT with a 95% CI. A Mann-Whitney test was performed in this analysis and P < 0.05 was considered statistically significant. *P < 0.05, **P < 0.01, ***P < 0.001, ****P < 0.0001. ns represents no statistical significance.

The effects of booster vaccination, gender, symptoms, and comorbidities on the level of Nab of SARS-CoV-2 variants were then analyzed. Nab titers against the WT strain, BA.5, XBB.1 and EG.5 variants were not significantly different between the CoronaVac and BBIBP-CorV (*P*>0.05) (**Figure 3A**). Our results showed that individuals who experienced Omicron infection within <12 months after booster vaccination exhibited higher Nab levels against WT, BA.5 and XBB.1 variants at 1 month post-infection (**Figure 3B**). We further analyzed the impact of comorbidities on Nab titers in elderly individuals. The results showed that the Nab levels were lower in patients with comorbidities compared to those without. Notably, the antibody titers were lower in patients with cardiovascular diseases than in those with diabetes, although this difference was not statistically significant. Additionally, the Nab levels against BA.5 and XBB.1 variants in patients with cardiovascular disease were significantly lower than those patients without comorbidities (*P* < 0.05) (**Figure 3C**). When antibody levels were analyzed in individuals with underlying diseases, the Nab titers of WT, BA.5, XBB.1, and EG.5 strains in the normal population were significantly higher than those in individuals with comorbidities (**Figure 3C**).

### 3.3 The characteristic of the memory B cells, Tfh cells, and Th17 cells in the elderly

**Figure 4:**
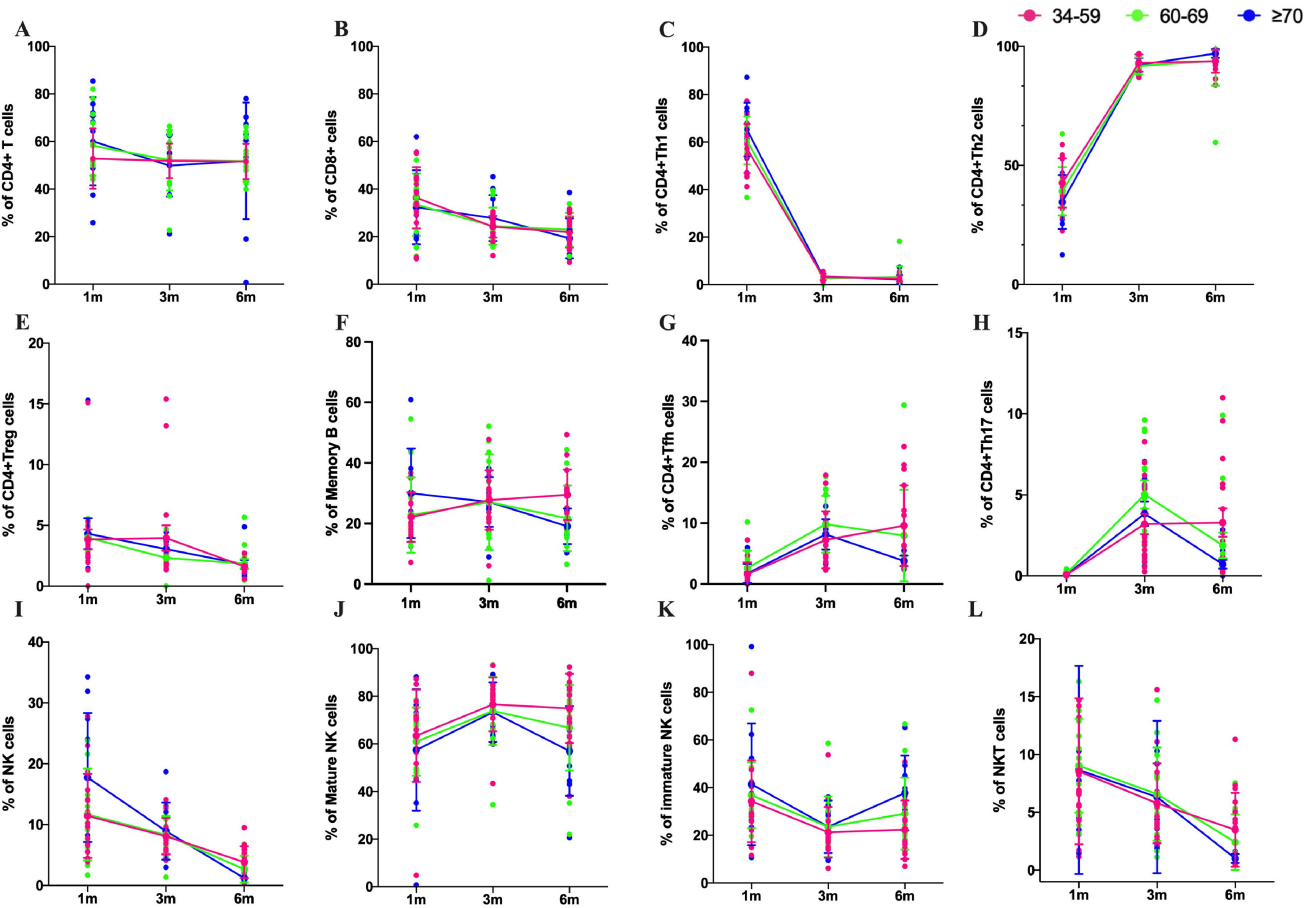
Subset changes of T cells, B cells and NK cells after Omicron BA.5 infection. The number of T cells, B cells and NK cells without peptide stimulation in middle-age in 34–59 years old (pink), the elderly over 60 years old (green), the elderly over 70 years old (blue) in 1m, 3m, and 6m pi, respectively. **(A, B)** Kinetics of CD4^+^ and CD8^+^T cells responses in three age groups. **(C, D)** Kinetics of Th1 and Th2 cells in three age groups. **(E)** Kinetics of CD4^+^Treg cells in three age groups. **(F)** Kinetics of memory B cells responses in three age groups. **(G)** Kinetics of Tfh cells responses in three age groups. **(H)** Kinetics of Th17 cells responses in three age groups. **(I)** Kinetics of NK cells responses in three age groups. **(J, K)** Kinetics of mature NK and immature NK cells responses in three age groups, respectively. **(L)** Kinetics of immature NKT cells responses in three age groups.

To understand the dynamic changes of the immune cells of the population after Omicron BA.5 infection, memory B cells, CD4^+^T cells, CD8^+^T cells, and CD4^+^T cell subset were detected at the above three time points. The results showed that the total number of total CD4^+^T cells remained stable at 1m, 3m and 6m pi and CD8^+^T cells showed a significant decreasing trend over time (**Figures 4A, B**). The number of Th1 cells decreased over time from 1m pi to 3m pi and then stabilized until 6m pi, whereas Th2 cells showed the opposite trend (**Figures 4C, D**). The number of CD4^+^Treg cells decreased continuously over time whether in older people or the middle-aged population (**Figure 4E**). However, the number of memory B cells in the elderly population decreased with the prolongation of post-infection time. Still, the number of memory B cells (MBC) in the middle-aged population continued to grow from 1m to 3m pi and remained relatively stable from 3m to 6m pi (**Figure 4F**). Our results also showed that the number of Tfh cells and Th17 cells continuously increased in a middle-aged population. However, the number of Tfh cells and Th17 cells showed a characteristic of first increasing from 1m to 3m pi and then decreasing from 3m to 6m pi in older people (**Figures 4G, H**).

NK cells and NKT cells, the main groups of immune cells involved in innate immunity, were also analyzed in this study. The proportion of NK cells decreased rapidly from 1m pi to 6m pi, with the fastest decline in people over 70 years of age (**Figure 4I**). The proportion of mature NK cells in the middle-aged population increased from 1m pi to 3m pi and remained stable from 3m pi to 6m pi. Mature NK cells in the older population showed a similar trend from 1m to 3m pi, but it decreased from 3m to 6m pi. Our results also showed that the proportion of mature NK cells was higher than in the middle-aged in the older people at each time point (**Figure 4J**). However, immature NK cells exhibited a completely opposite trend (**Figure 4K**). The proportion of NKT cells in all age groups showed a decreasing trend (**Figure 4L**).

### 3.4 SARS-CoV-2-specific cellular immunity of the elderly after infection

#### 3.4.1 Activation of CD4^+^/CD8^+^T cells decreased in older people

**Figure 5:**
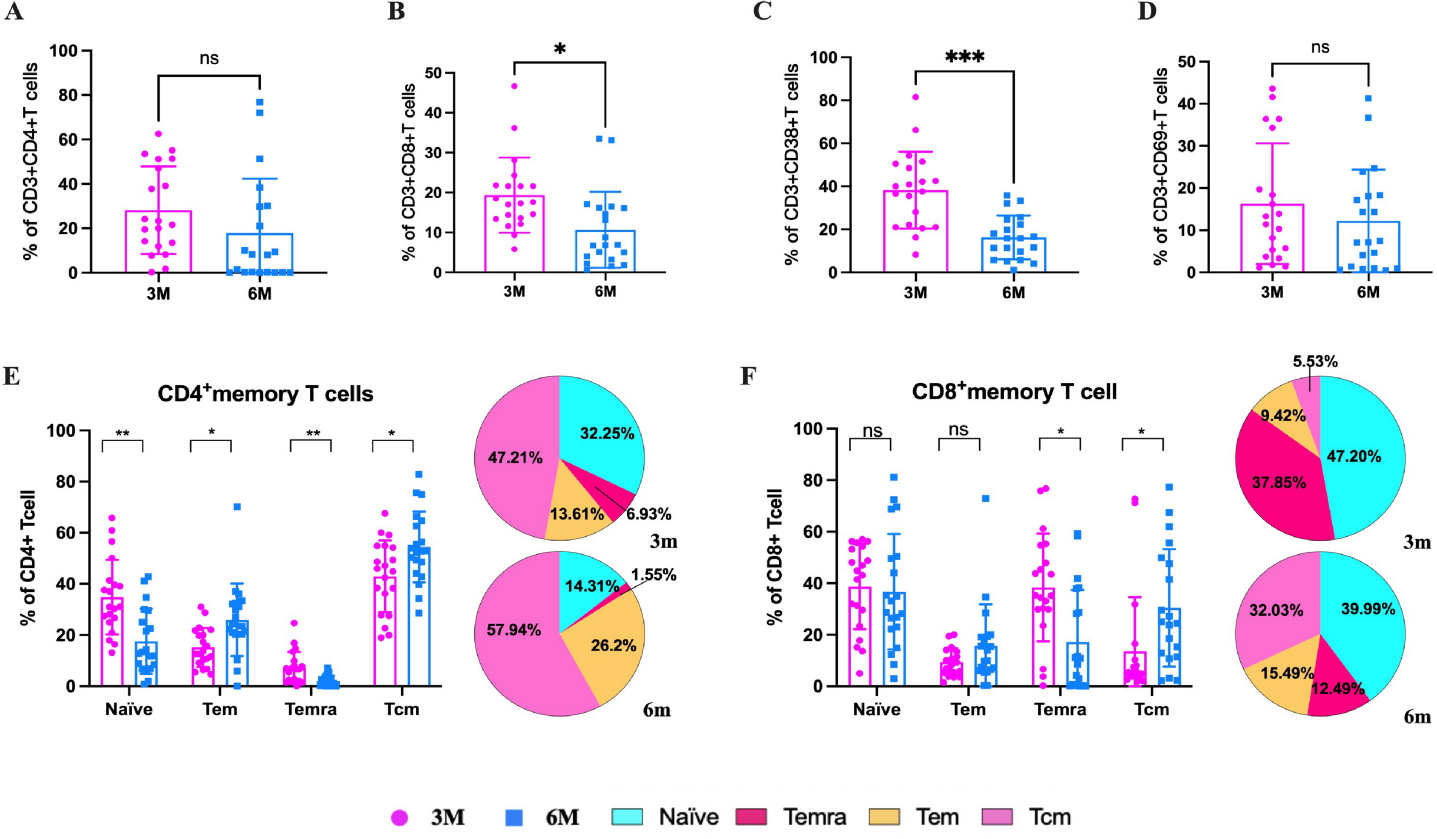
SARS-CoV-2-specific T cell responses in elderly individuals at 3m and 6m pi. After SARS-CoV-2 spike peptide stimulation, **(A)** specific CD4^+^ T cells and **(B)** specific CD8^+^ T cells in elderly individuals at 3m and 6 m pi. **(C)** CD38^+^ T cells and **(D)** CD69^+^ T cells levels in elderly individuals at 3m and 6 m pi. **(E)** specific CD4^+^ T cell and **(F)** CD8^+^ T cell memory phenotypes: naïve, central memory T cells, effector memory T cells, terminally differentiated effector T cells. The bar chart shows dynamic changes from 3m to 6m. The pie chart shows the proportion of four memory T cell phenotypes at 3m and 6m. A Mann-Whitney test was performed in this analysis and P < 0.05 was considered statistically significant. *P < 0.05, **P < 0.01, ***P < 0.001, ****P < 0.0001. ns represents no statistical significance.

To characterize the specific cellular immune effects and immune memory response in older people, PBMCs from the elderly at 3m pi and 6m pi were stimulated with the peptide pool of S protein of BA.5 variants. After stimulation, both CD4^+^ T cells and CD8^+^ T cells showed a decreasing trend from 3m to 6m pi (**Figures 5A, B**). Then, T-cell activation was identified through detection of CD38 and CD69 markers. The result found that the proportion of both CD38^+^ T cells and CD69^+^ T cells decreased from 3m to 6m pi (**Figures 5C, D**). To investigate the memory phenotypes of SARS-CoV-2-specific CD4^+^ and CD8^+^ T cells, the proportion of the naïve (CD45RA^+^CCR7^+^), central memory (Tcm, CD45RA^−^CCR7^+^), effector memory (Tem, CD45RA^−^CCR7^−^), and late effector (Temra, CD45RA^+^CCR7^−^) subsets were determined by detection of both CD45RA and CCR7. The results showed that the specific Tcm, Tem, naïve, and Temra cells of CD4^+^ T cells in the elderly accounted for 47.21%, 13.61%, 32.25%, and 6.93% in 3m pi, respectively (**Figure 5E**). Compared with Tcm and Tem cells at 3m pi, both Tcm and Tem cells of the elderly increased significantly at 6m pi (57.94%, 26.20%), whereas both naïve and Temra cells decreased at 6m pi (14.31%, 1.55%) (*P*<0.05) (**Figure 5E**).

Similar to the results above, for CD8*^+^* T cells, Tcm cells increased from 5.53% to 32.02%, and Tem cells increased from 9.42% to 15.49% from 3m to 6m pi in older people (**Figure 5F**). However, the Temra cells decreased from 37.85% to 12.49%, and naïve cells decreased from 47.20% to 39.99% from 3m to 6m pi, respectively (**Figure 5F**).

#### 3.4.2 MBC, unswitched/switched MBC decreased and DNBC increased in elderly

**Figure 6:**
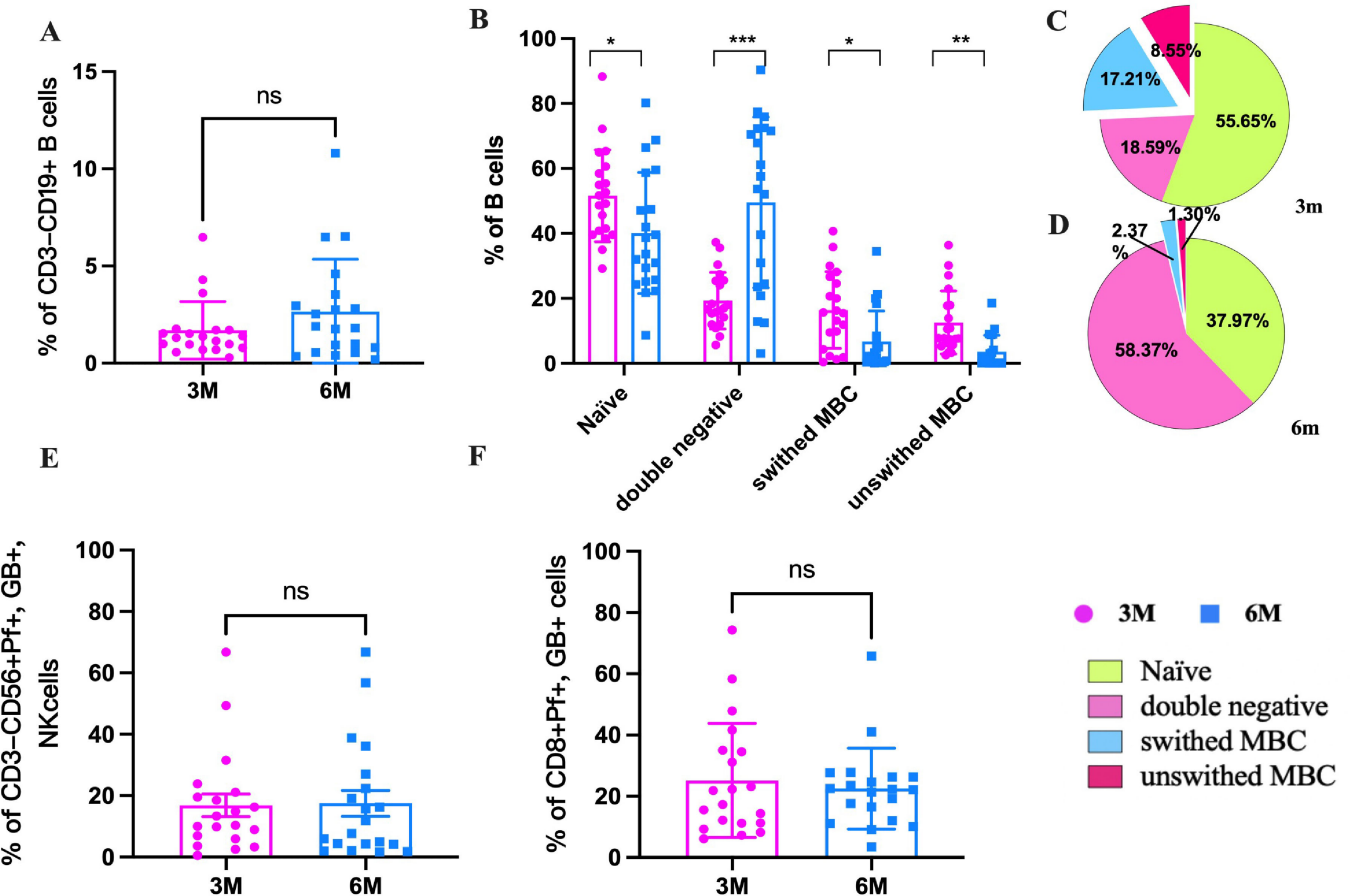
The frequencies of SARS-CoV-2-specific memory B cells, NK cells and CTL cells in the elderly at 3m and 6m pi. **(A)** The changes in B cells at 3m, 6m pi in elderly convalescent patients. **(B)** Classification of B cell subtypes in elderly convalescent patients at 3m and 6m pi. **(C)** Proportional distribution of B cell subtypes at 3m pi in elderly convalescent patients. **(D)** Proportional distribution of B cell subtypes at 6m pi in elderly convalescent patients. **(E)** The changes of mature NK cells (Perforin^+^ GranzymB^+^, CD56^+^) at 3m, 6m pi in elderly convalescent patients. **(F)** The changes of CTL cells (Perforin+ GranzymB^+^, CD8^+^) at 3m, 6m pi in elderly convalescent patients. A Mann-Whitney test was performed in this analysis and P < 0.05 was considered statistically significant. *P < 0.05, **P < 0.01, ***P < 0.001, ****P < 0.0001. ns represents no statistical significance.

The function of NK cells and B cells was then assessed following stimulation with the peptide pool. The four subsets of B cells, namely naïve/transitional B cells (CD27^-^IgD^+^), unswitched memory B cells (unswitched MBC, CD27^+^IgD^+^), switched memory B cells (switched MBC, CD27^+^IgD^-^), and double negative B cells (CD27^-^IgD^-^), were evaluated according to the different expression levels of CD19, CD27, and IgD. The results showed that the B cells (CD19^+^) increased slightly from 3m to 6m pi (*P*>0.05) (**Figure 6A**). Comparing the B cell subsets of convalescents at 3m and 6m pi, it was found that naïve B cells, unswitched MBC, and switched MBC were significantly lower in 6m pi than in 3m pi, but double negative B cells (DNBC) were considerably higher than that in 3m pi (**Figure 6B**). The naïve B cells made up the majority of B cells in 3m pi, whereas DNBC made up the majority of B cells in 6m pi. Furthermore, the frequency of unswitched MBC was lower than switched MBC in both 3m pi and 6m pi (**Figures 6C, D**). Our results also showed that the Perforin^+^, Granzyme^+^ of NK cells and CD8^+^T cells also decreased slightly after stimulation (**Figures 6E, F**).

#### 3.4.3 PD-1^+^/CTLA-4^+^ CD4^+^/CD8^+^ T-cell increased in older people

**Figure 7:**
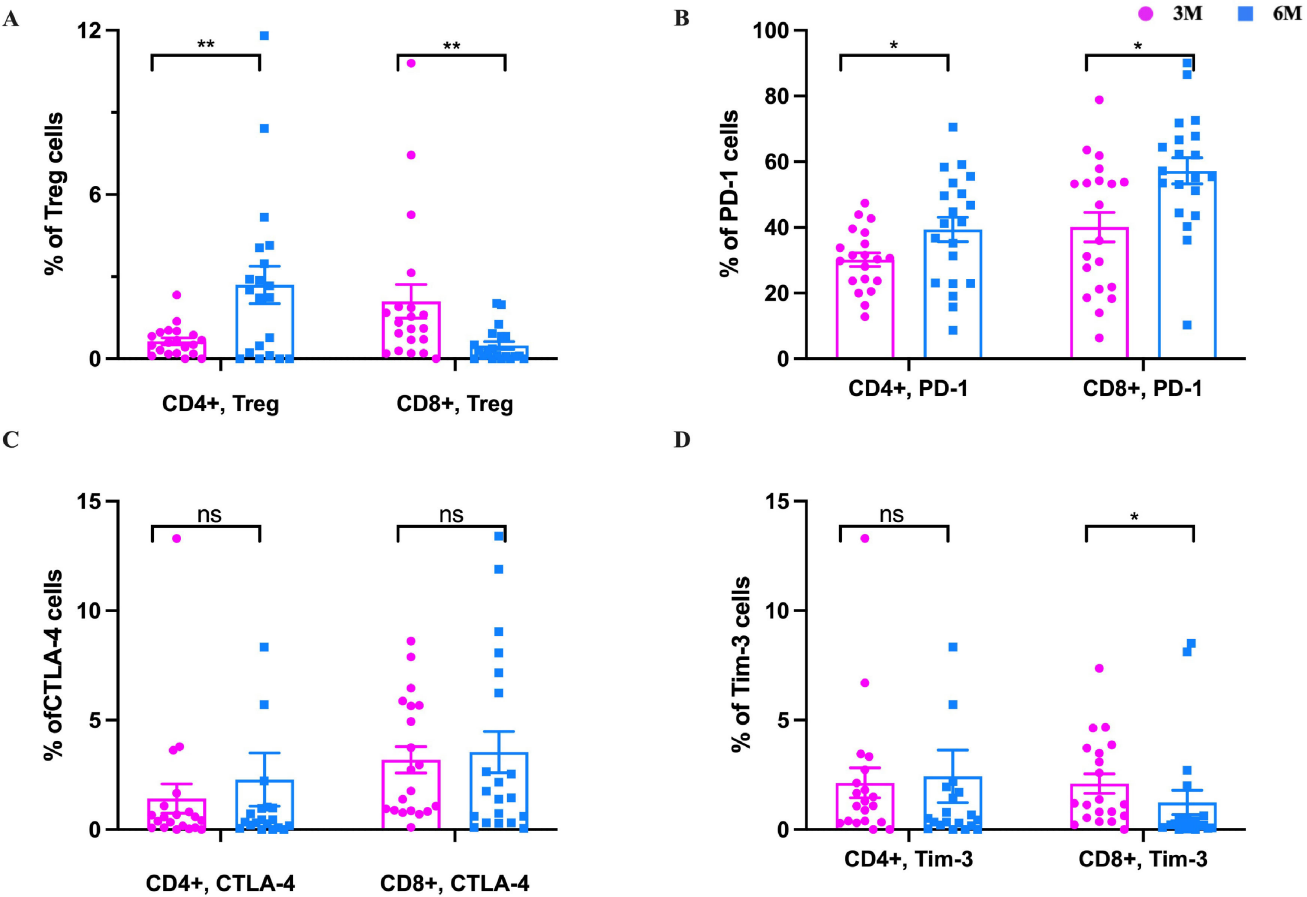
The changes of Treg cells and immune checkpoint molecules in the elderly. After SARS-CoV-2 spike peptide stimulation, **(A)** The changes of CD4^+^ Treg and CD8^+^ Treg cells at 3m, 6m pi in elderly convalescent patients. **(B)** The changes of CD4^+^PD-1^+^ and CD8^+^PD-1^+^ cells at 3m, 6m pi in elderly convalescent patients. **(C)** The changes of CD4^+^TIM-3^+^ and CD8^+^TIM-3^+^ cells at 3m, 6m pi in elderly convalescent patients. **(D)** The changes of CD4^+^CTLA-4^+^ and CD8^+^CTLA-4^+^ cells at 3m, 6m pi in elderly convalescent patients. A Mann-Whitney test was performed in this analysis and P < 0.05 was considered statistically significant. *P < 0.05, **P < 0.01, ***P < 0.001, ****P < 0.0001. ns represents no statistical significance.

Immune regulation determines not only the occurrence of an immune response caused by a viral infection but also determines the strength of the immune response. Regulatory T cells (Tregs) are the leading immunosuppressive cell group that maintains the immune balance of the body. Our results showed that CD4^+^ Tregs cells increased significantly from 3m to 6m pi, while CD8^+^ Tregs cells decreased significantly in 3m to 6m pi in older people (*P*<0.05) (**Figure 7A**). Immune checkpoint (ICP) is another type of immunosuppressive molecule that can regulate immune responses and prevent normal tissue damage and destruction. The common ICP molecules include programmed cell death protein 1 (PD-1), cytotoxic T-lymphocyte-associated protein 4 (CTLA-4), T cell immunoglobulin and mucin-domain containing 3 (Tim-3) (28). To understand the effect of ICP molecules in the elderly after BA.5 infection, PD-1^+^ (CD279), CTLA-4^+^ (CD152), and Tim-3^+^ (CD366) of CD4^+^ and CD8^+^ T cells were detected in this study. The results showed that PD-1^+^ of CD4^+^ and CD8^+^ T cells were significantly increased from 3m to 6m pi (**Figure 7B**). CTLA-4^+^ (CD152) cells of CD4^+^ and CD8^+^ T-cell also increased from 3m to 6m pi (*P*>0.05) (**Figure 7C**). Additionally, Tim-3^+^ cells of CD4^+^ T cells slightly increased, while Tim-3^+^ cells of CD8^+^ T-cell significantly decreased from 3m to 6m pi (**Figure 7D**).

In a 3m pi follow up study of elderly individuals with long COVID, six participants reported having at least one persistent symptom that lasted for ≥2 months. Further analysis revealed that the PD-1, CTLA-4, and Tim-3, were elevated in elderly subjects with long COVID symptoms compared to those without, even if there is no statistical difference for the majority. Although the majority of these differences did not reach statistical significance (**Supplementary Figure 3**).

## 4 Discussion

Older people are highly vulnerable to SARS-CoV-2 and its variants, mainly due to their poor antibody response (29). Therefore, it is crucial to determine whether the immune response to SARS-CoV-2 and its variants is preserved after Omicron BA.5 infection. To our knowledge, this study is the first to describe the dynamic changes in specific humoral and cellular immunity to Omicron BA.5 in elderly individuals.

Our study found that the Nab titers to WT and BA.5 were obviously higher than those of XBB.1 and EG.5 from 1m to 6m after BA.5 infection. The results indicated that the individuals infected with the SARS-CoV-2 variant had a stimulated immune memory response to the WT strain and showed higher immune protection against the BA.5 variant, but there was a poor cross-reaction against XBB.1 and EG.5 variant, which is consistent with the previously reported studies (30–33). This likely reflects immune evasion driven by key mutations in the spike receptor-binding domain (RBD). Specifically, F456L and F486P substitutions in XBB.1 variant alter antibody-binding interfaces through structural remodeling (34), while the F456L/Q52H mutations in EG.5 enhance ACE2 binding and simultaneously evade common neutralizing antibodies (35). These findings were consistent with structural evidence indicating that F456L mutation alone reduces antibody neutralization potency by >20-fold (34). Prior to the emergence of Omicron variants, the effectiveness of CoronaVac booster vaccination in preventing symptomatic COVID-19 among adults was 78.8% (36), Vaccine-induced neutralizing antibody titers against the Omicron variant were 16.5-fold lower compared to those against the prototype strain (37). Our findings also showed that the Nab titer of the WT strain at the time of booster vaccination that < 12 m was significantly higher than that of ≥12 m. Similar to early results (31, 38), the Nab titer of the WT strain and the Omicron BA.5 strain was weaker in older people. This may suggest that the elderly are more susceptible to infection with new variant strains. We also found that fever, cough, and fatigue were the most obvious symptoms among the older population following Omicron BA.5 infection.

Similar to our research findings in the middle-aged population, after symptomatic SARS-CoV-2 infection, the frequency of MBC peaks between 4 and 5 months (20). However, MBCs frequencies decreased from 3m to 6m pi in the elderly individuals. MBC are a subtype of B cells that form in germinal center after an initial infection and play an important role in producing an accelerated and stronger antibody-mediated immune response (also known as secondary immune response) in the event of a subsequent infection. In the human immune system, the number of MBC is generally higher than that of naïve B cells in the peripheral blood, especially in the elderly population (39). However, in our study, the number of MBC was lower than that of naïve B cells in 3m pi. The decrease in MBC may be due to their transformation into plasma cells that produce a large amount of antibodies. Our results also showed that both unswitched MBC and switched MBC decreased to a lower level, and Naïve B cells decreased from 55.85% to 37.97%. However, the double negative B cells (DNBC), as late-stages memory cells, increased from 15.59% in 3m pi to 58.37% in 6m pi. DNBC can secrete inflammatory mediators such as TNF-α, IL-6, and IL-8 (40–43), which may be involved in the persistent inflammatory response in the elderly population. From our results, it appears that the expansion of DNBCs is not related to antibody production, but may be related to the persistent increase in cytokines. This expansion of such cells can also be shown in critically ill patients with COVID-19 exhibiting characteristics of lupus-like autoimmune disease (42).

MBC have a long lifespan and remain in a dormant state until they reencounter antigens. As exposed to exogenous antigens again, Tfh cells immediately reactivate MBC, which differentiate into plasma cells capable of secreting antibodies. Similar to the trend of unswitched MBC and switched MBC, the number of Tfh cells in the elderly population showed a peak in the third month after infection and a decrease in the sixth month after infection. This result demonstrated the synergistic effect between Tfh cells and MBC after SARS-CoV-2 infection. The number of Th17 and Tfh cells in middle-aged people continued to increase with prolonged infection time. Th17 cells are associated with protective immune responses in specific viral infection models (44, 45). However, in other cases, they have been shown to promote the pathogenesis of viral diseases (44, 46). From our research results, the Th17 cell continued to increase after infection in middle-aged people, suggesting that it does not promote the prognosis of patients with COVID-19 infection. The functional relevance of the Th17 response to COVID-19 requires further investigation.

Our results showed that, in elderly individuals, the proportions of naïve and Temra cells in CD4^+^/CD8^+^T cells decline, whereas those of Tcm and Tem cells rise. Notably, the proportion of naïve cells in CD4^+^T cells is significantly lower than that of Tcm cells. After T-cell division and differentiation, memory T cells and effector T cells are formed, respectively. Memory T cells can exist in the human body for months or even decades, preventing the body from being invaded again by the relevant pathogens. The validity period of some memory T cells is very short, such as influenza, hepatitis B, etc., but some are lifetime, such as smallpox (47–49). Memory T cells are typically divided into three main groups: Tcm, Tem, and Temra cells. This study showed that Tcm of CD4^+^ T cells from older people showed a significantly higher proliferation frequency from 47.21% to 57.94% over time. Tcm cells of CD8^+^T cells increased rapidly from 5.53% 3m pi to 32.03% 6m pi. This may be due to the rapid transformation of naïve T cells, prompting the body to respond quickly. Temra cells decreased from 6.93% to 1.55% for CD4^+^T cells, and Temra cells of CD8^+^T cells rapidly reduced from 37.85% to 12.49%. The results indicate that at the early stage (3m pi), Temra cells may play some roles in the early inflammation of COVID-19 infection. Our results still showed that the Tem cells of CD4^+^ cells increased from 13.61% to 26.20%, and the Tem cells of CD8^+^ cells also increased from 9.42% to 15.49%, respectively. This suggests that the Tcm cells may be one of important immune protection in the elderly population. A decreased proportion of naïve CD4^+^T cells indicates that the immune response to new antigens in the elderly population is limited. It is difficult to establish long-term and adequate immune protection, and they may be more prone to reinfection.

Naïve CD4^+^ T cells differentiate into several effector cell subsets based on their function and the cytokines they release. The major subsets are Th1, Th2, Th17, Treg and Tfh cells (50). Th1 cells are essential for the host’s defense toward intracellular pathogens (51), while Th2 cells mediate the activation and maintenance of the antibody-mediated immune response against extracellular parasites, bacteria, allergens, and toxins (52). Similar to typical research findings, our study showed that both the elderly and middle-aged populations exhibited opposite trends in Th1 and Th2 cell responses over time. Within 6 months after COVID-19 infection, NK cells and NKT cells decreased with the extension of post-infection time in both the elderly and middle-aged populations. The results suggest that the antiviral response of the body infected with COVID-19 gradually returns to normal. Our results also showed that the number of mature NK cells in the elderly population was slightly lower than that in the middle-aged population.

Tregs are key immunosuppressive cells that migrate to areas of inflammation and help suppress the inflammatory response (53, 54). We found that the number of CD4^+^Treg cells increased with time after infection, while the number of CD8^+^Treg cells showed the opposite trend. PD-1, Tim-3, and CTLA-4 of ICP molecules are associated with T cell depletion (28, 55, 56). At 6 months after infection, the number of cells expressing PD-1^+^, CTLA-4^+^ in CD4^+^ and CD8^+^ cells were higher than that at 3 months after infection; A previous study demonstrates that PD-1 expressing SARS-CoV-2 specific CD4^+^ T cells were exhausted (46). In our study, PD-1 showed an increasing trend in the elderly after infection. Thus PD-1 expressed on effector SARS-CoV-2 specific T cells may gradually act as an exhaustion marker in the long-term recovery phase over time. CTLA-4 also increased during recovery, consistent with other studies (56). This further suggests that these older people do not maintain persistent immune function after infection. Divergent Treg dynamics and rising immune exhaustion markers: unveiling the older people’s post-infection immune vulnerability.

This study has several limitations. Firstly, the small sample size of elderly participants will limit the scalability of our results. Secondly, the lack of a control cohort for young people makes it impossible to exclude age-specific immune features.

## 5 Conclusion

There are few studies on adaptive immune cohorts of Omicron variants in the elderly after the COVID-19 pandemic. Here we find that the Nab titers showed higher immune protection against the WT strains and BA.5 variant after BA.5 infection, but poor cross-reaction against XBB.1 and EG.5, which means that these populations are more likely to be infected with either XBB.1 or EG.5 again. The decrease of the MBC, unswitched/switched MBC and CD69^+^/CD38^+^T cells, the differences in Tfh and Th17 cell changes between the elderly population and the middle-aged population, as well as the increase of CD4^+^/CD8^+^PD-1^+^ T cells, CTLA-4^+^CD4^+^/CD8^+^T cells and DNBC, suggest that the elderly exhibit a reduced persistent immune response to BA.5 infection. In addition, elderly individuals with comorbidities have an increased risk of developing serious illnesses when reinfected with XBB.1 and EG.5 variants. Vaccination of the elderly population against newly emerging variants is one of the key strategies to reduce the risk of reinfection for this vulnerable group.

## Data Availability

The raw data supporting the conclusions of this article will be made available by the authors, without undue reservation.

## References

[1] World Health Organization. WHO Coronavirus (COVID-19) Dashboard with Vaccination Data 2023 . Available online at: https://data.who.int/dashboards/covid19 (Accessed May 8, 2025).

[2] Carabelli AM Peacock TP Thorne LG Harvey WT Hughes J Consortium C-GU. SARS-CoV-2 variant biology: immune escape, transmission and fitness. Nat Rev Microbiol. (2023) 21:162–77. doi: 10.1038/s41579-022-00841-7 PMC9847462 36653446

[3] Ortega MA Garcia-Montero C Fraile-Martinez O Colet P Baizhaxynova A Mukhtarova K. Recapping the features of SARS-coV-2 and its main variants: status and future paths. J Pers Med. (2022) 12(6):995. doi: 10.3390/jpm12060995 PMC9225183 35743779

[4] Ao D He X Hong W Wei X. The rapid rise of SARS-CoV-2 Omicron subvariants with immune evasion properties: XBB.1.5 and BQ.1.1 subvariants. MedComm (2020). (2023) 4:e239. doi: 10.1002/mco2.239 36938325 PMC10015854

[5] Allen H Tessier E Turner C Anderson C Blomquist P Simons D. Comparative transmission of SARS-CoV-2 Omicron (B.1.1.529) and Delta (B.1.617.2) variants and the impact of vaccination: national cohort study, England. Epidemiol Infect. (2023) 151:e58. doi: 10.1017/S0950268823000420 36938806 PMC10125873

[6] Pan Y Wang L Feng Z Xu H Li F Shen Y. Characterisation of SARS-CoV-2 variants in Beijing during 2022: an epidemiological and phylogenetic analysis. Lancet. (2023) 401:664–72. doi: 10.1016/S0140-6736(23)00129-0 PMC9949854 36773619

[7] Chinese Center for Disease Control and Prevention. Situation of SARS-CoV-2 infection in China . Available online at: https://www.Chinacdc.cn/jksj/xgbdyq/202503/t20250324_305223.html (Accessed May 8, 2025).

[8] Nikolich-Zugich J Knox KS Rios CT Natt B Bhattacharya D Fain MJ. SARS-CoV-2 and COVID-19 in older adults: what we may expect regarding pathogenesis, immune responses, and outcomes. Geroscience. (2020) 42:505–14. doi: 10.1007/s11357-020-00186-0 PMC7145538 32274617

[9] Bencivenga L Rengo G Varricchi G. Elderly at time of COronaVIrus disease 2019 (COVID-19): possible role of immunosenescence and malnutrition. Geroscience. (2020) 42:1089–92. doi: 10.1007/s11357-020-00218-9 PMC7308600 32578073

[10] Canaday DH Carias L Oyebanji OA Keresztesy D Wilk D Payne M. Reduced BNT162b2 messenger RNA vaccine response in severe acute respiratory syndrome coronavirus 2 (SARS-coV-2)-naive nursing home residents. Clin Infect Dis. (2021) 73:2112–5. doi: 10.1093/cid/ciab447 PMC8240817 33993265

[11] Collier DA Ferreira I Kotagiri P Datir RP Lim EY Touizer E. Age-related immune response heterogeneity to SARS-CoV-2 vaccine BNT162b2. Nature. (2021) 596:417–22. doi: 10.1038/s41586-021-03739-1 PMC8373615 34192737

[12] Wang J Tong Y Li D Li J Li Y. The impact of age difference on the efficacy and safety of COVID-19 vaccines: A systematic review and meta-analysis. Front Immunol. (2021) 12:758294. doi: 10.3389/fimmu.2021.758294 34938287 PMC8685545

[13] Lang PO Govind S Bokum AT Kenny N Matas E Pitts D. Immune senescence and vaccination in the elderly. Curr Top Med Chem. (2013) 13:2541–50. doi: 10.2174/15680266113136660181 24066892

[14] Soegiarto G Wulandari L Purnomosari D Dhia Fahmita K Ikhwan Gautama H Tri Hadmoko S. Hypertension is associated with antibody response and breakthrough infection in health care workers following vaccination with inactivated SARS-CoV-2. Vaccine. (2022) 40:4046–56. doi: 10.1016/j.vaccine.2022.05.059 PMC9135674 35660034

[15] Ashrafian F Bagheri Amiri F Bavand A Zali M Sadat Larijani M Ramezani A. A comparative study of immunogenicity, antibody persistence, and safety of three different COVID-19 boosters between individuals with comorbidities and the normal population. Vaccines (Basel). (2023) 11(8):1376. doi: 10.3390/vaccines11081376 PMC10459403 37631944

[16] Zhao XJ Liu XL Liang YM Zhang S Liu T Li LB. Epidemiological characteristics and antibody kinetics of elderly population with booster vaccination following both Omicron BA.5 and XBB waves in China. J Med Virol. (2024) 96:e29640. doi: 10.1002/jmv.29640 38699969

[17] Cao Y Jian F Wang J Yu Y Song W Yisimayi A. Imprinted SARS-CoV-2 humoral immunity induces convergent Omicron RBD evolution. Nature. (2023) 614:521–9. doi: 10.1038/s41586-022-05644-7 PMC9931576 36535326

[18] Wajnberg A Amanat F Firpo A Altman DR Bailey MJ Mansour M. Robust neutralizing antibodies to SARS-CoV-2 infection persist for months. Science. (2020) 370:1227–30. doi: 10.1126/science.abd7728 PMC7810037 33115920

[19] Feng C Shi J Fan Q Wang Y Huang H Chen F. Protective humoral and cellular immune responses to SARS-CoV-2 persist up to 1 year after recovery. Nat Commun. (2021) 12:4984. doi: 10.1038/s41467-021-25312-0 34404803 PMC8370972

[20] Dan JM Mateus J Kato Y Hastie KM Yu ED Faliti CE. Immunological memory to SARS-CoV-2 assessed for up to 8 months after infection. Science. (2021) 371(6529):eabf4063. doi: 10.1126/science.abf4063 33408181 PMC7919858

[21] Rydyznski Moderbacher C Ramirez SI Dan JM Grifoni A Hastie KM Weiskopf D. Antigen-specific adaptive immunity to SARS-coV-2 in acute COVID-19 and associations with age and disease severity. Cell. (2020) 183:996–1012 e1019. doi: 10.1016/j.cell.2020.09.038 33010815 PMC7494270

[22] Liao M Liu Y Yuan J Wen Y Xu G Zhao J. Single-cell landscape of bronchoalveolar immune cells in patients with COVID-19. Nat Med. (2020) 26:842–4. doi: 10.1038/s41591-020-0901-9 32398875

[23] Zhou R To KK Wong YC Liu L Zhou B Li X. Acute SARS-coV-2 infection impairs dendritic cell and T cell responses. Immunity. (2020) 53:864–877 e865. doi: 10.1016/j.immuni.2020.07.026 32791036 PMC7402670

[24] Fong CH Zhang X Chen LL Poon RW Chan BP Zhao Y. Effect of vaccine booster, vaccine type, and hybrid immunity on humoral and cellular immunity against SARS-CoV-2 ancestral strain and Omicron variant sublineages BA.2 and BA.5 among older adults with comorbidities: a cross sectional study. EBioMedicine. (2023) 88:104446. doi: 10.1016/j.ebiom.2023.104446 36706582 PMC9874281

[25] Taira N Toguchi S Miyagi M Mori T Tomori H Oshiro K. Altered pre-existing SARS-CoV-2-specific T cell responses in elderly individuals. Clin Immunol Commun. (2022) 2:6–11. doi: 10.1016/j.clicom.2021.12.001 38621014 PMC8694817

[26] Guan X Huang Q Dong M Li M Xie H Wei X. SARS-CoV-2-specific antibody and T-cell immunity in convalescents after infection wave in Beijing in late 2022. J Infect. (2023) 87:413–9. doi: 10.1016/j.jinf.2023.08.010 37652314

[27] Bond K Nicholson S Lim SM Karapanagiotidis T Williams E Johnson D. Evaluation of serological tests for SARS-coV-2: implications for serology testing in a low-prevalence setting. J Infect Dis. (2020) 222:1280–8. doi: 10.1093/infdis/jiaa467 PMC7454699 32761124

[28] Retnakumar SV Chauvin C Bayry J. The implication of anti-PD-1 therapy in cancer patients for the vaccination against viral and other infectious diseases. Pharmacol Ther. (2023) 245:108399. doi: 10.1016/j.pharmthera.2023.108399 37001736

[29] Xia S Jiao F Wang L Yu X Lu T Fu Y. SARS-CoV-2 Omicron XBB subvariants exhibit enhanced fusogenicity and substantial immune evasion in elderly population, but high sensitivity to pan-coronavirus fusion inhibitors. J Med Virol. (2023) 95:e28641. doi: 10.1002/jmv.28641 36890632

[30] Uraki R Ito M Furusawa Y Yamayoshi S Iwatsuki-Horimoto K Adachi E. Humoral immune evasion of the omicron subvariants BQ.1.1 and XBB. Lancet Infect Dis. (2023) 23:30–2. doi: 10.1016/S1473-3099(22)00816-7 PMC9729000 36495917

[31] Wang H Xue Q Zhang H Yuan G Wang X Sheng K. Neutralization against Omicron subvariants after BA.5/BF.7 breakthrough infection weakened as virus evolution and aging despite repeated prototype-based vaccination(1). Emerg Microbes Infect. (2023) 12:2249121. doi: 10.1080/22221751.2023.2249121 37668156 PMC10524800

[32] Yang J Hong W Lei H He C Lei W Zhou Y. Low levels of neutralizing antibodies against XBB Omicron subvariants after BA.5 infection. Signal Transduct Target Ther. (2023) 8:252. doi: 10.1038/s41392-023-01495-4 37336889 PMC10279763

[33] Liu S Liang Z Nie J Gao WB Li X Zhang L. Sera from breakthrough infections with SARS-CoV-2 BA.5 or BF.7 showed lower neutralization activity against XBB.1.5 and CH.1.1. Emerg Microbes Infect. (2023) 12:2225638. doi: 10.1080/22221751.2023.2225638 37313604 PMC10339773

[34] Yisimayi A Song W Wang J Jian F Yu Y Chen X. Repeated Omicron exposures override ancestral SARS-CoV-2 immune imprinting. Nature. (2024) 625:148–56. doi: 10.1038/s41586-023-06753-7 PMC10764275 37993710

[35] Esmaeilzadeh A Ebrahimi F Jahani Maleki A Siahmansouri A. EG.5 (Eris) and BA.2.86 (Pirola) two new subvariants of SARS-CoV-2: a new face of old COVID-19. Infection. (2024) 52:337–43. doi: 10.1007/s15010-023-02146-0 38170417

[36] Jara A Undurraga EA Zubizarreta JR Gonzalez C Pizarro A Acevedo J. Effectiveness of homologous and heterologous booster doses for an inactivated SARS-CoV-2 vaccine: a large-scale prospective cohort study. Lancet Glob Health. (2022) 10:e798–806. doi: 10.1016/S2214-109X(22)00112-7 PMC9034854 35472300

[37] Wang K Jia Z Bao L Wang L Cao L Chi H. Memory B cell repertoire from triple vaccinees against diverse SARS-CoV-2 variants. Nature. (2022) 603:919–25. doi: 10.1038/s41586-022-04466-x PMC8967717 35090164

[38] Chen Y Klein SL Garibaldi BT Li H Wu C Osevala NM. Aging in COVID-19: Vulnerability, immunity and intervention. Ageing Res Rev. (2021) 65:101205. doi: 10.1016/j.arr.2020.101205 33137510 PMC7604159

[39] Duggal NA. Reversing the immune ageing clock: lifestyle modifications and pharmacological interventions. Biogerontology. (2018) 19:481–96. doi: 10.1007/s10522-018-9771-7 PMC6223743 30269199

[40] Cancro MP. Age-associated B cells. Annu Rev Immunol. (2020) 38:315–40. doi: 10.1146/annurev-immunol-092419-031130 31986068

[41] Frasca D. Senescent B cells in aging and age-related diseases: Their role in the regulation of antibody responses. Exp Gerontol. (2018) 107:55–8. doi: 10.1016/j.exger.2017.07.002 PMC5754260 28687479

[42] Woodruff MC Ramonell RP Nguyen DC Cashman KS Saini AS Haddad NS. Extrafollicular B cell responses correlate with neutralizing antibodies and morbidity in COVID-19. Nat Immunol. (2020) 21:1506–16. doi: 10.1038/s41590-020-00814-z PMC7739702 33028979

[43] Bartleson JM Radenkovic D Covarrubias AJ Furman D Winer DA Verdin E. SARS-coV-2, COVID-19 and the ageing immune system. Nat Aging. (2021) 1:769–82. doi: 10.1038/s43587-021-00114-7 PMC8570568 34746804

[44] Acharya D Wang P Paul AM Dai J Gate D Lowery JE. Interleukin-17A promotes CD8+ T cell cytotoxicity to facilitate west nile virus clearance. J Virol. (2017) 91(1):e01529-16. doi: 10.1128/JVI.01529-16 27795421 PMC5165211

[45] Wang X Chan CC Yang M Deng J Poon VK Leung VH. A critical role of IL-17 in modulating the B-cell response during H5N1 influenza virus infection. Cell Mol Immunol. (2011) 8:462–8. doi: 10.1038/cmi.2011.38 PMC4012931 21946434

[46] Ma WT Yao XT Peng Q Chen DK. The protective and pathogenic roles of IL-17 in viral infections: friend or foe? Open Biol. (2019) 9:190109. doi: 10.1098/rsob.190109 31337278 PMC6685926

[47] Spitaels J Roose K Saelens X. In fl uenza and Memory T Cells: How to Awake the Force. Vaccines (Basel). (2016) 4:33. doi: 10.3390/vaccines4040033 27754364 PMC5192353

[48] Wang RX Boland GJ van Hattum J de Gast GC. Long-term persistence of T cell memory to HBsAg after hepatitis B vaccination. World J Gastroenterol. (2004) 10:260–3. doi: 10.3748/wjg.v10.i2.260 PMC4717016 14716835

[49] Kunzli M Masopust D. CD4(+) T cell memory. Nat Immunol. (2023) 24:903–14. doi: 10.1038/s41590-023-01510-4 PMC10343737 37156885

[50] Murphy KM Ouyang W Farrar JD Yang J Ranganath S Asnagli H. Signaling and transcription in T helper development. Annu Rev Immunol. (2000) 18:451–94. doi: 10.1146/annurev.immunol.18.1.451 10837066

[51] Graham MB Dalton DK Giltinan D Braciale VL Stewart TA Braciale TJ. Response to influenza infection in mice with a targeted disruption in the interferon gamma gene. J Exp Med. (1993) 178(5):1725–32. doi: 10.1084 jem.178.5.1725 10.1084/jem.178.5.1725 PMC2191239 8228818

[52] Chaplin DD. Overview of the immune response. J Allergy Clin Immunol. (2010) 125:S3–23. doi: 10.1016/j.jaci.2009.12.980 20176265 PMC2923430

[53] Gladstone DE Kim BS Mooney K Karaba AH D'Alessio FR. Regulatory T cells for treating patients with COVID-19 and acute respiratory distress syndrome: two case reports. Ann Intern Med. (2020) 173:852–3. doi: 10.7326/L20-0681 PMC7370819 32628535

[54] Mortezaee K Majidpoor J. Cellular immune states in SARS-CoV-2-induced disease. Front Immunol. (2022) 13:1016304. doi: 10.3389/fimmu.2022.1016304 36505442 PMC9726761

[55] Govender M Hopkins FR Goransson R Svanberg C Shankar EM Hjorth M. T cell perturbations persist for at least 6 months following hospitalization for COVID-19. Front Immunol. (2022) 13:931039. doi: 10.3389/fimmu.2022.931039 36003367 PMC9393525

[56] Hou H Zhang Y Tang G Luo Y Liu W Cheng C. Immunologic memory to SARS-CoV-2 in convalescent COVID-19 patients at 1 year postinfection. J Allergy Clin Immunol. (2021) 148:1481–1492 e1482. doi: 10.1016/j.jaci.2021.09.008 34536418 PMC8440318

