# Supplementary material for: Reduced immune response to SARS-CoV-2 infection in the elderly after 6 months
Source: Front Immunol. 2025 May 9;16:1596065. doi: 10.3389/fimmu.2025.1596065 (PMC12098630; doi:10.3389/fimmu.2025.1596065)
Supplement: Supplementary file 1 [file DataSheet1.docx]

Supplementary Material

# 1 Supplementary Figures

For more information on Supplementary Material and for details on the different file types accepted, please see [here](https://www.frontiersin.org/guidelines/author-guidelines#supplementary-material).


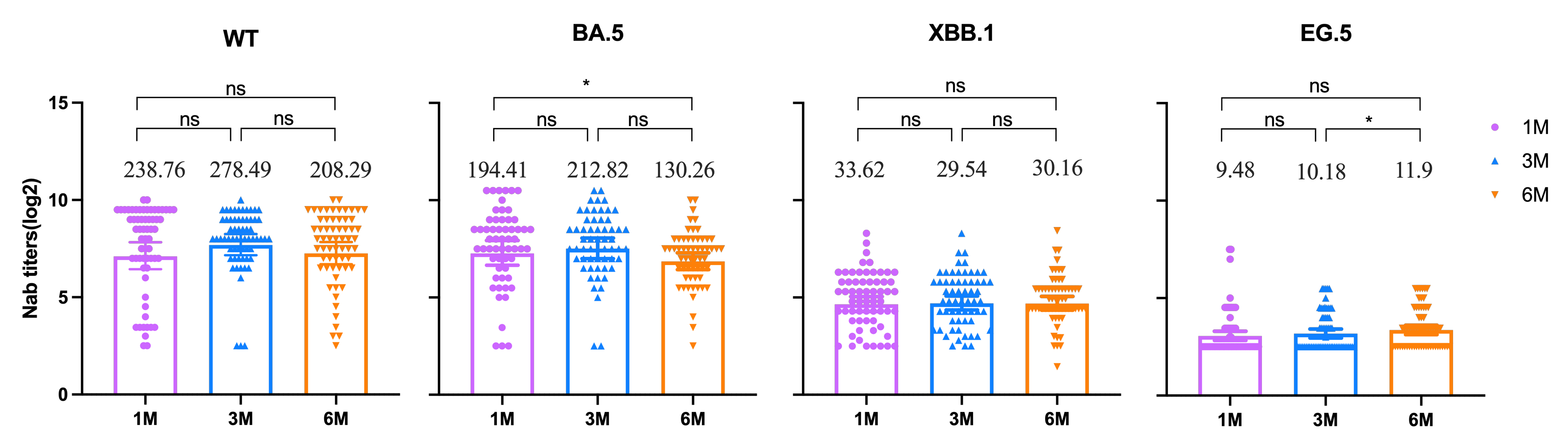


**Supplementary Figure 1. The Nab titers against the WT strain BA.5, XBB.1, EG.5 variant in 1m, 3m and 6m p.i.**

NAb titers of SARS-CoV-2 in the 58 participants in 1m, 3m, and 6m pi. The GMT values were presented at the top of the figure. A Wilcoxon matched-pairs signed rank test was performed in this analysis and p < 0.05 was considered statistically significant. *p < 0.05, **p < 0.01, ***p < 0.001, ****p < 0.0001.


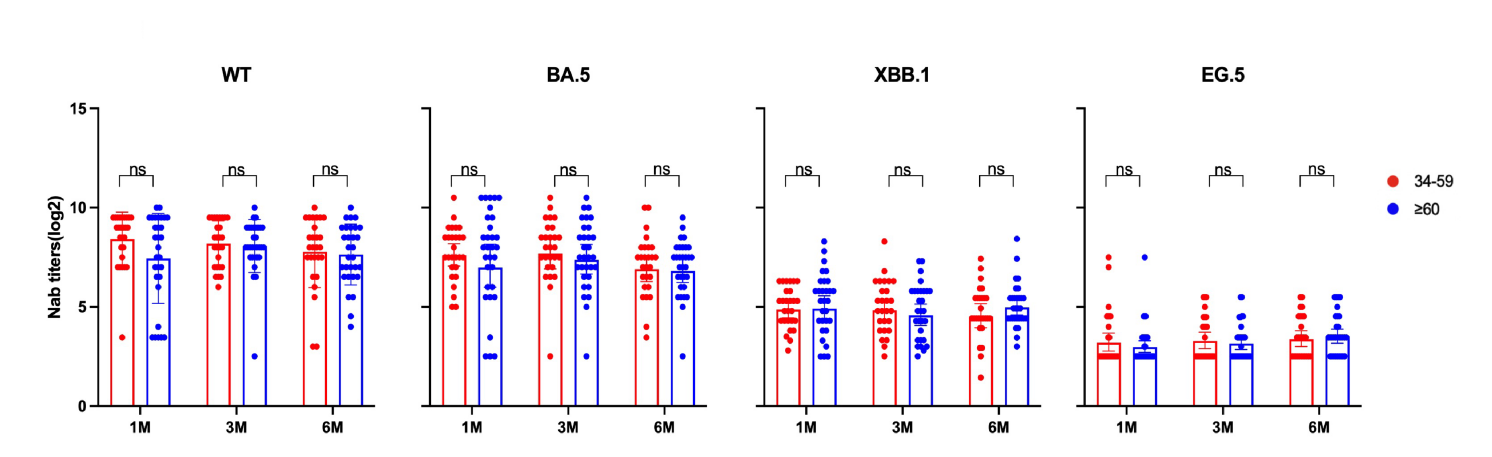


**Supplementary Figure 2. The influence of age on Nab levels of WT, BA.5, XBB.1, and EG.5**.

NAb titers against WT and Omicron BA.5, XBB.1, EG.5 variants in the middle-age and the elderly. The geometric mean titer (GMT) values were presented at the top of the figure. A Mann-Whitney test was performed in this analysis and p < 0.05 was considered statistically significant. *p < 0.05, **p < 0.01, ***p < 0.001, ****p < 0.0001.


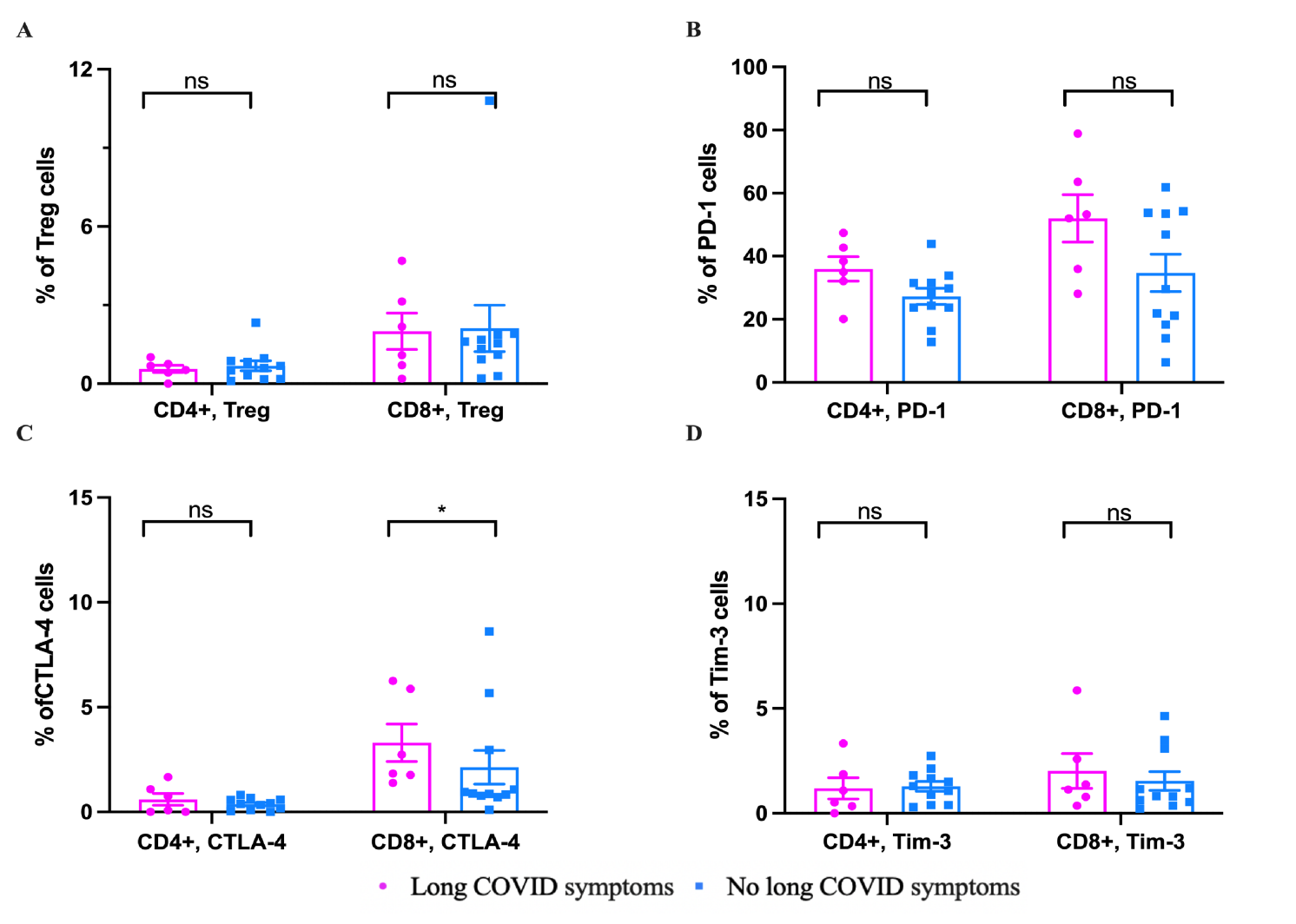


**Supplementary Figure 3. Changes in Treg cells and immune checkpoint molecules in older people with long COVID symptoms.**

A Wilcoxon matched-pairs signed rank test was performed in this analysis and p < 0.05 was considered statistically significant. *p < 0.05, **p < 0.01, ***p < 0.001, ****p < 0.0001.
